# [Retracted] Uvangoletin induces mitochondria-mediated apoptosis in HL-60 cells *in vitro* and *in vivo* without adverse reactions of myelosuppression, leucopenia and gastrointestinal tract disturbances

**DOI:** 10.3892/or.2025.9015

**Published:** 2025-11-03

**Authors:** Zhuanzhen Zheng, Zhenhua Qiao, Rong Gong, Yalin Wang, Yiqun Zhang, Yanping Ma, Li Zhang, Yujin Lu, Bo Jiang, Guoxia Li, Chunxia Dong, Wenliang Chen

Oncol Rep 35: 1213–1221, 2016; DOI: 10.3892/or.2015.4443

Following the publication of this paper, it was drawn to the Editor's attention by a concerned reader that, concerning the flow cytometric plots featured in Fig. 3A-C on p. 1215, certain groups of data points appeared more similar to each other than might be expected.

The authors were asked for an explanation to account for these concerns, but the Editorial Office did not receive a reply. Therefore, the Editor of *Oncology Reports* has decided that this paper should be retracted from the journal on account of a lack of confidence in the presented data. The Editor apologizes to the readership for any inconvenience caused.

